# Supplementary material for: CellContrast: Reconstructing spatial relationships in single-cell RNA sequencing data via deep contrastive learning
Source: Patterns (N Y). 2024 Jul 9;5(8):101022. doi: 10.1016/j.patter.2024.101022 (PMC11368686; doi:10.1016/j.patter.2024.101022)
Supplement: Document S1. Figures S1–S18 and Tables S1–S8 [file mmc1.pdf]

Patterns, Volume *5*

Supplemental information

CellContrast: Reconstructing spatial relationships in single-cell RNA sequencing data via deep contrastive learning

Shumin Li, Jiajun Ma, Tianyi Zhao, Yuran Jia, Bo Liu, Ruibang Luo, and Yuanhua Huang

Contents

1. Supplementary Figures 1
2. Supplementary Tables 19

1. Supplementary Figures

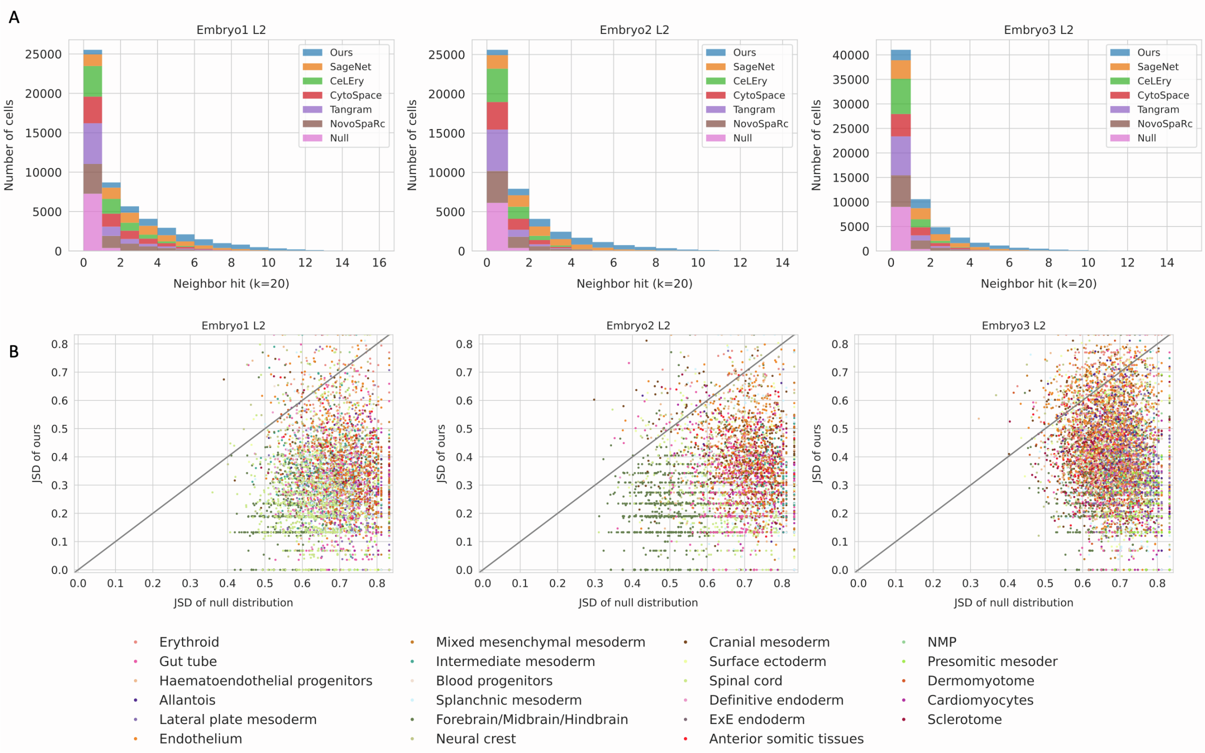

Figure S1. Evaluation of neighbor reconstruction in mouse gastrulation cells using SeqFISH ST reference. A, Distribution of hit number for reconstructed nearest 20 cell neighbors. B, Comparison of JSD distribution between our method and null distribution (randomly shuffled locations of the testing sample).

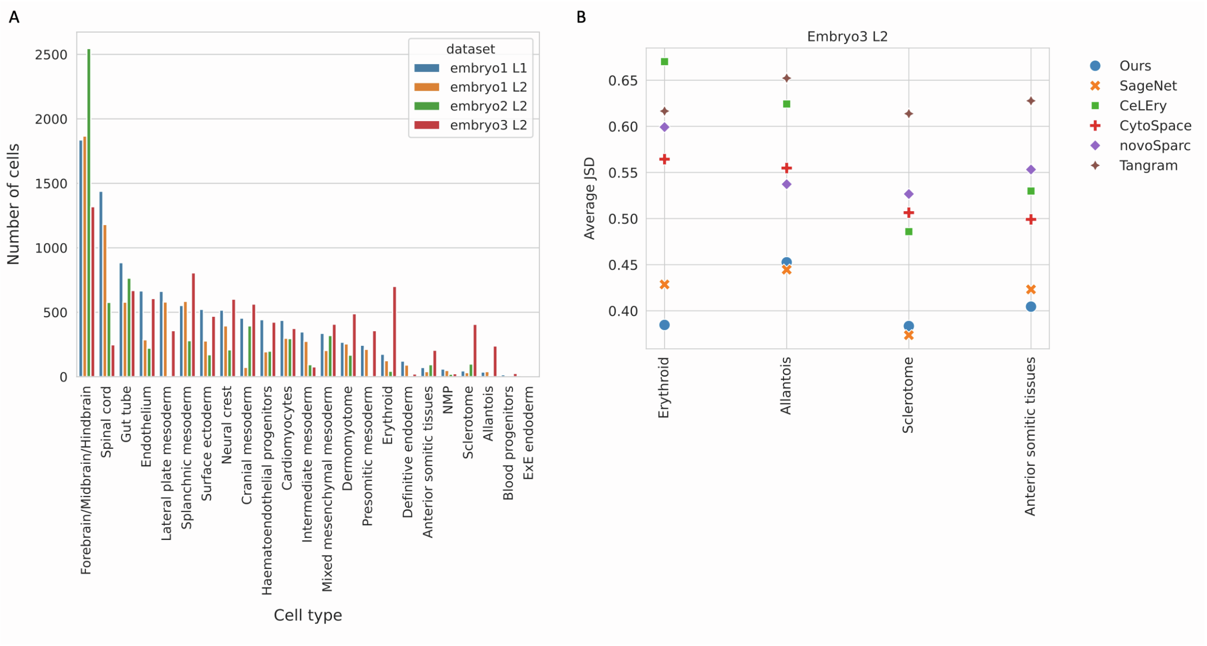

Figure S2. Analysis of cell type distribution and JSD in SeqFISH training and testing datasets. A, the cell type distribution for the training (embryo1 L1) and 3 testing datasets (L2 of embryo 1,2,3). B, The average JSD distribution for the four imbalanced cell-types between training and the testing sample embryo3 L2.

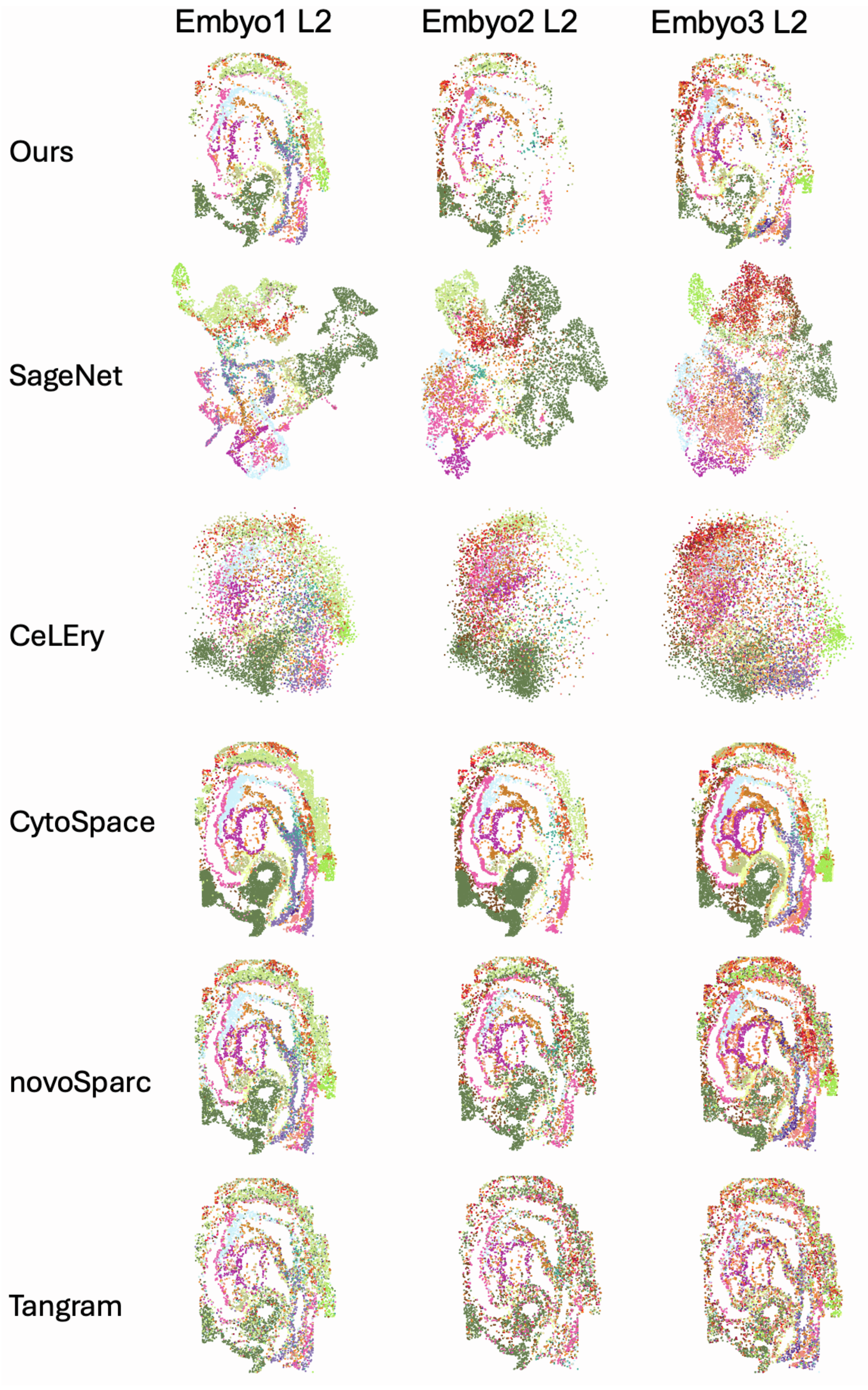

Figure S3. Spatial reconstruction results for L2 of embryo1,2,3 using embryo1 L2 of SeqFISH data as the reference. Noted that for SageNet produces pairwise cell-cell distance instead of coordinates, we generate the 2D distribution of cells using UMAP based on the distance matrix.

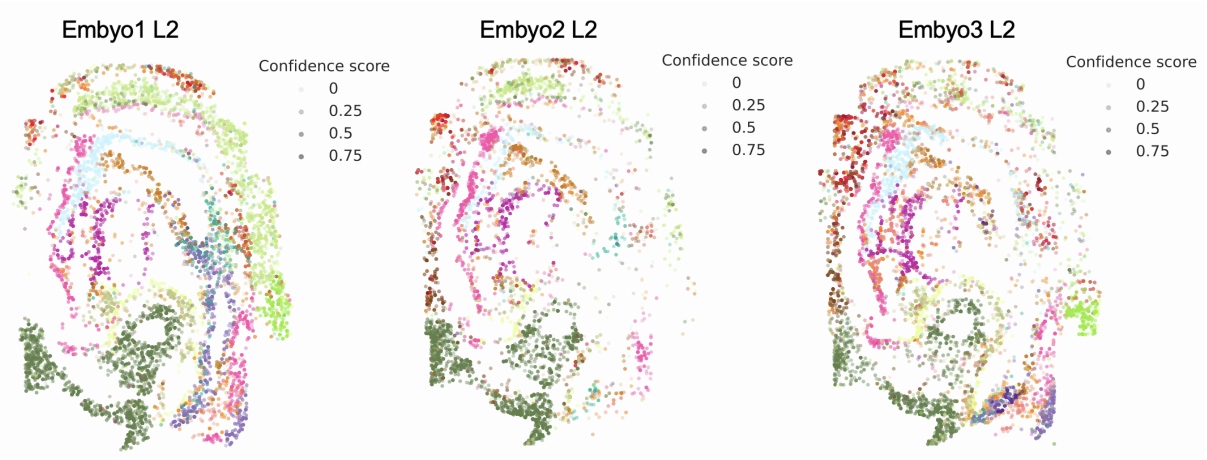

Figure S4. Spatial reconstruction of 3 query seqFISH samples mapped to reference locations of embryo1 L1. Cells are colored based on their cell type, and the transparency of each cell is determined by CellContrast's confidence score.

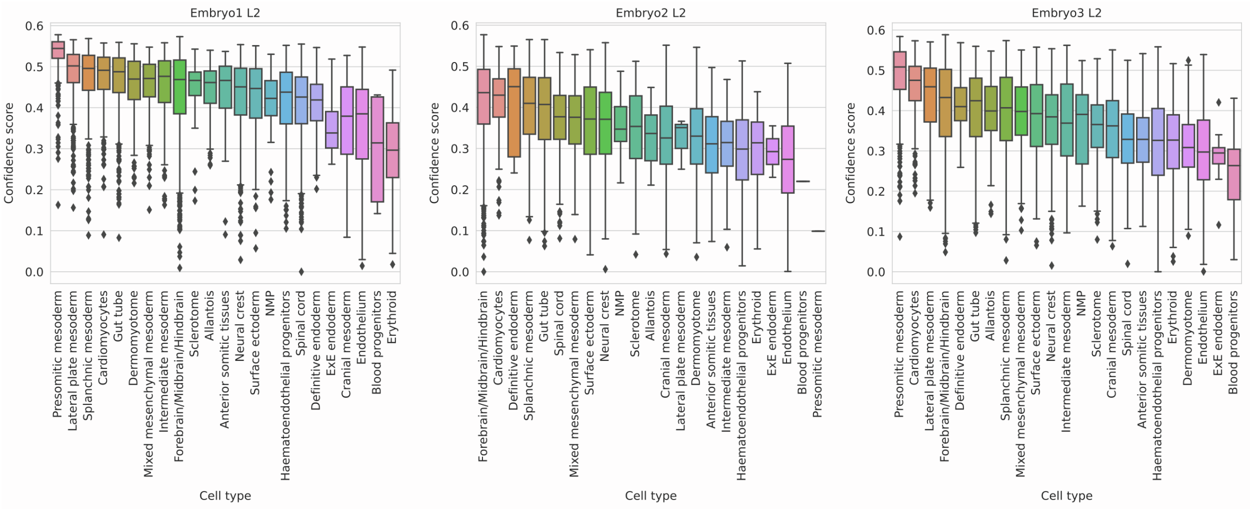

Figure S5. Distribution of mapping confidence scores for cell types. The three query seqFISH samples are mapped to the reference locations of embryo1 L1 by CellContrast's SC-ST mapping.

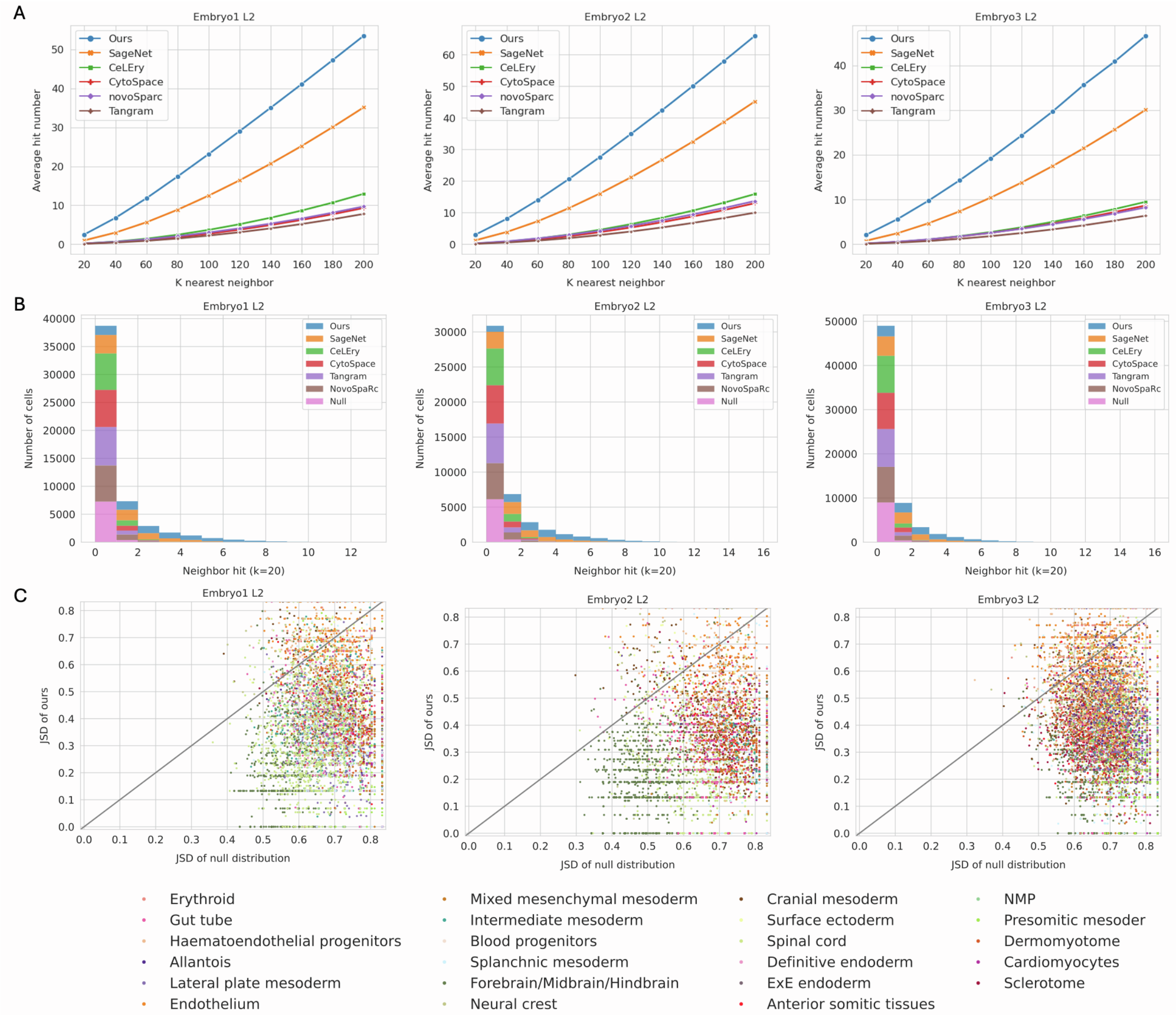

Figure S6. Evaluation of neighbor reconstruction in mouse gastrulation cells using array-based ST reference. A. Average hit number with varying *k* nearest neighbors for 3 testing samples: L2 of embryo 1,2, 3. B. Distribution of hit number for reconstructed nearest 20 cell neighbors. C. Comparison of JSD distribution between our method and null distribution (randomly shuffled locations of the testing sample).

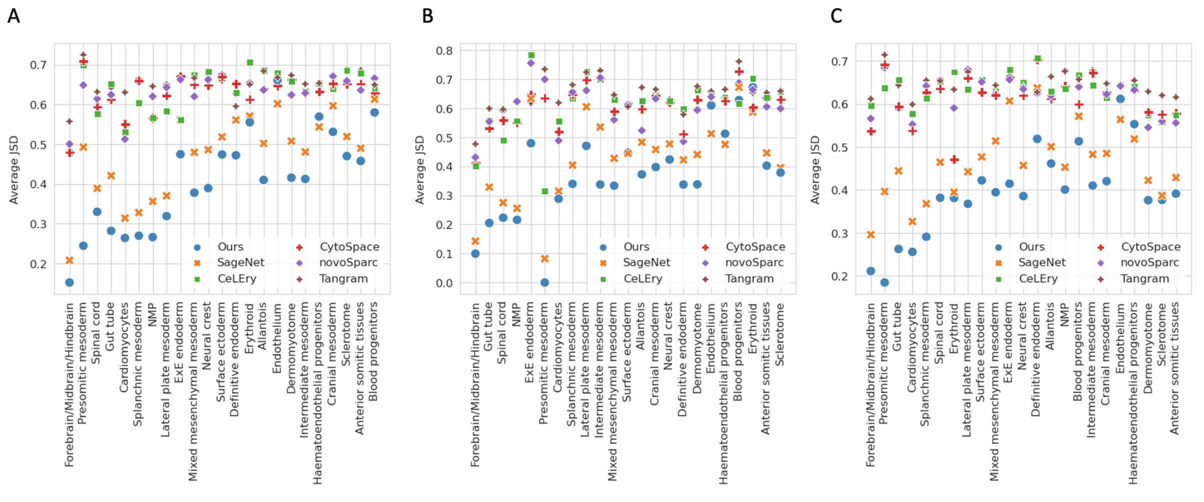

Figure S7. Evaluation of local neighborhood cell-type heterogeneity in mouse gastrulation cells using array-based ST reference. A, Jessen-Shannon distance of cell types for testing dataset embryo1 L2. B, Jessen-Shannon distance of cell types for testing dataset embryo2 L2. C, Jessen-Shannon distance of cell types for testing dataset embryo3 L2.

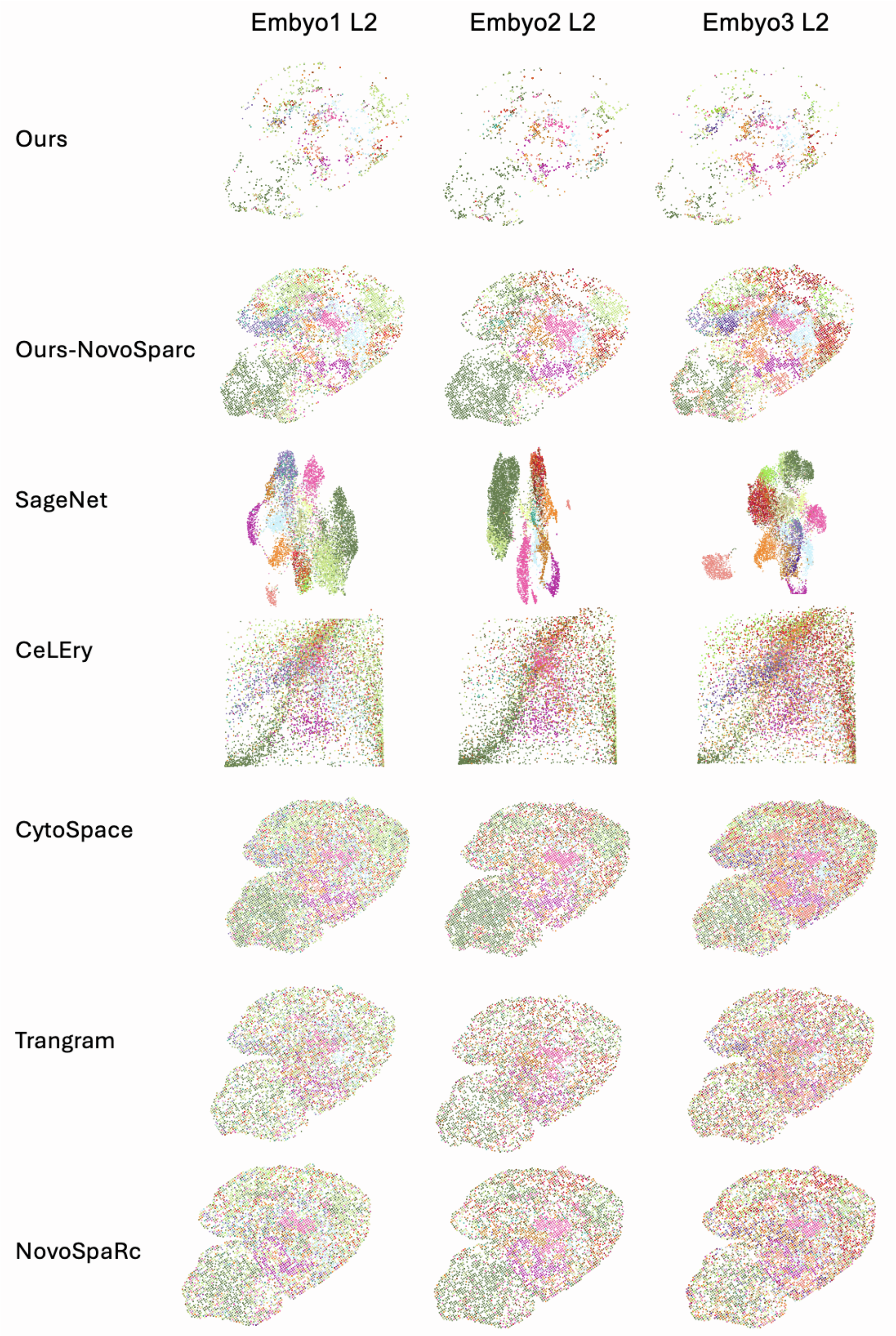

Figure S8. Spatial reconstruction results for L2 of embryo 1, 2, 3 using Stereo-Seq data as the reference. Noted that for SageNet produces predicted pairwise cell-cell distance instead of coordinates, we generate the 2D distribution of cells using umap based on the distance matrix. We also combined our latent representation of cells as the input to NovoSpaRc, marked as “ours-NovoSparc”. It is an effective way for 2D visualization but might not be the optimal for down-stream tasks. When the gap between the ST and SC is large, we recommend using our estimated SC-SC distances.

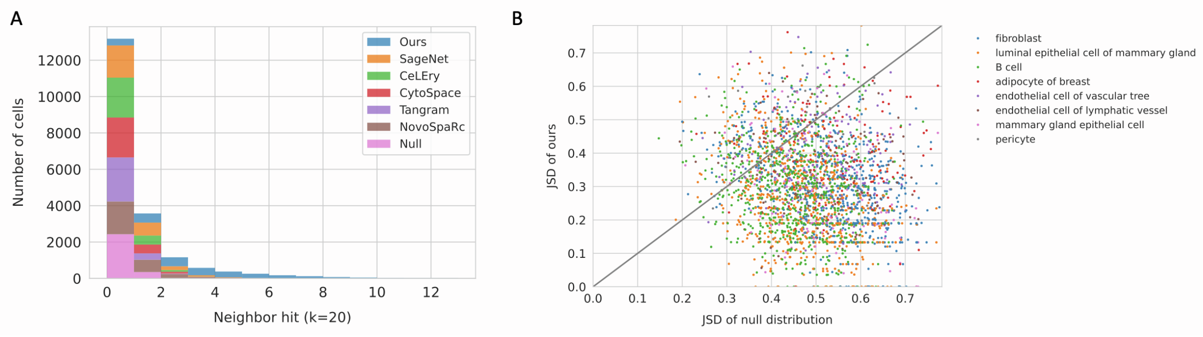

Figure S9. The local reconstruction performance comparison with Null distribution for human breast 10X Visium data. A, Distribution of hit number for reconstructed the nearest 20 cell neighbors. B, Comparison of JSD distribution between our method and null distribution (randomly shuffled locations of the testing sample).

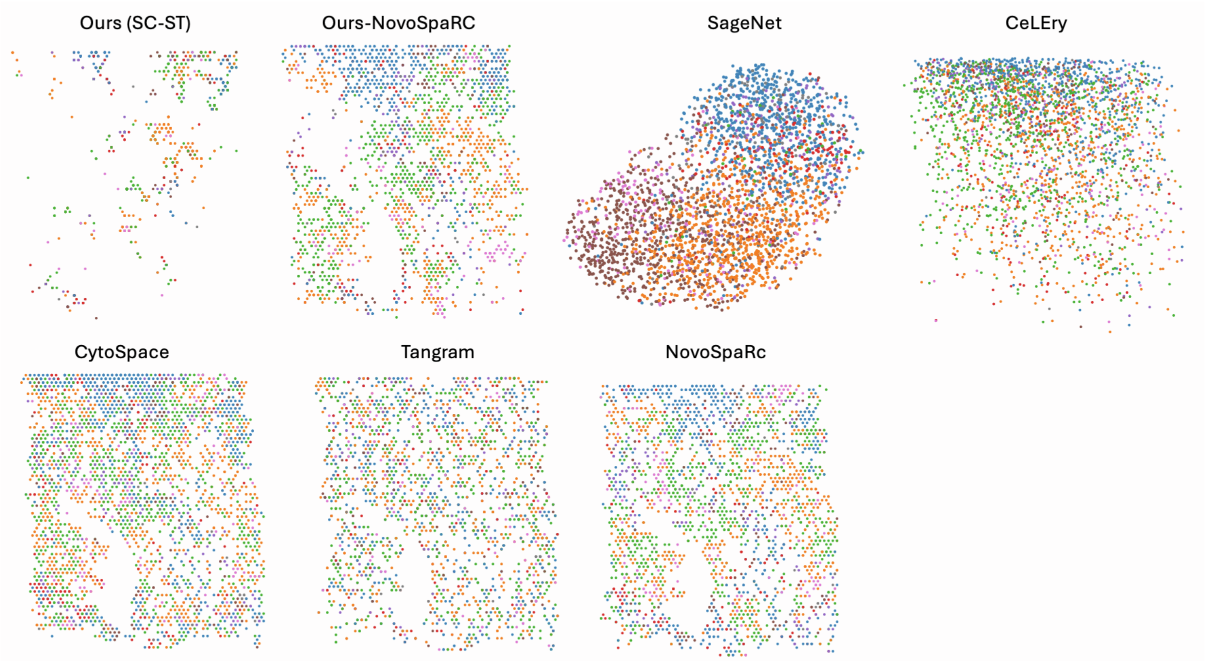

Figure S10. Spatial reconstruction results for results for human lung sample generated from 10X Visium. Noted that for SageNet produces predicted pairwise cell-cell distance instead of coordinates, we generate the 2D distribution of cells using umap based on the distance matrix. We also combined our latent representation of cells as the input to NovoSpaRc, marked as “ours-NovoSpaRc”. It is an effective way for 2D visualization but might not be the optimal for down-stream tasks. When the gap between the ST and SC is large, we recommend using our estimated SC-SC distances.

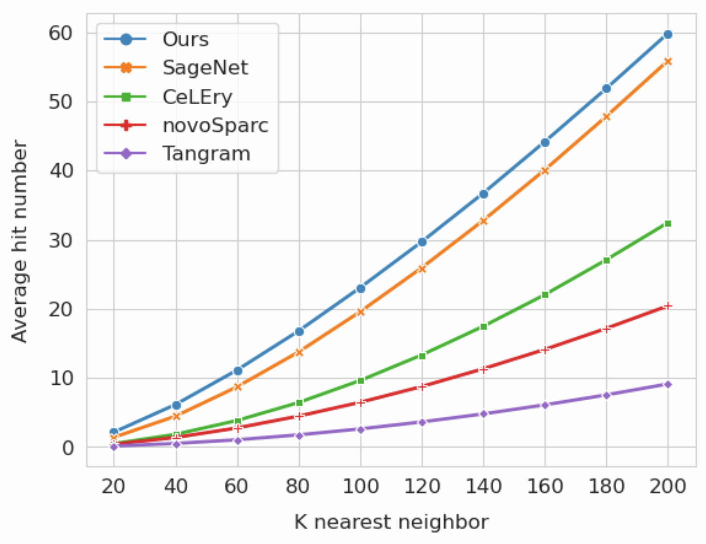

Figure S11. Evaluation of neighbor reconstruction in mouse brain cells generated by MERCOPE. Noted that CytoSpace was excluded from the analysis due to the absence of cell type annotations, as cell type information is a required parameter for its single-cell mode.

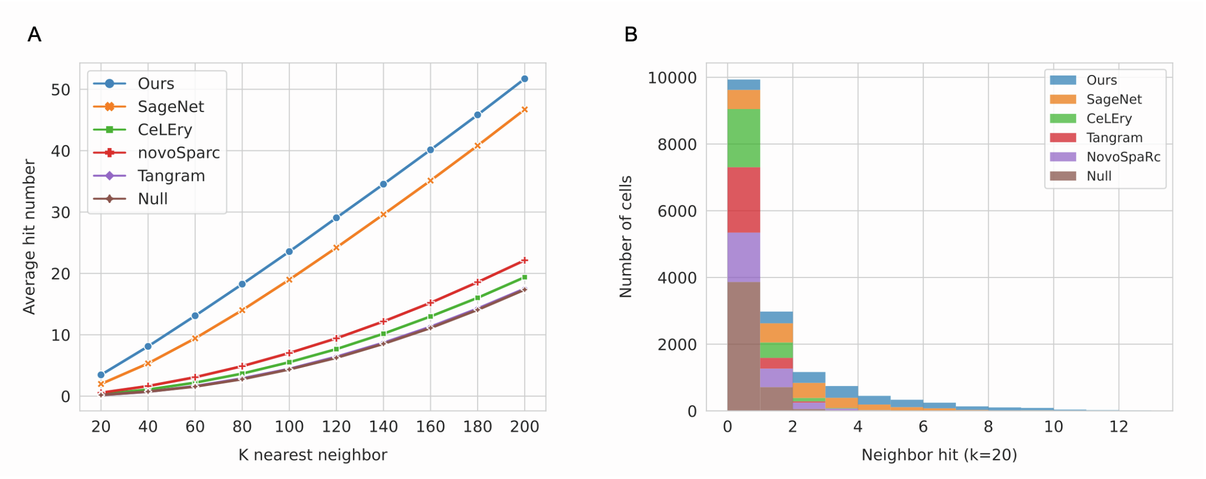

Figure S12. Local reconstruction performance for human lung samples generated by 10X Visium. Noted that CytoSpace was excluded from the analysis due to the absence of cell type annotations, as cell type information is a required parameter. A, Evaluation of cell neighbor reconstruction by average neighbor hit within varying k nearest neighbors. B, Distribution of neighbor hit within nearest 20 neighbors.

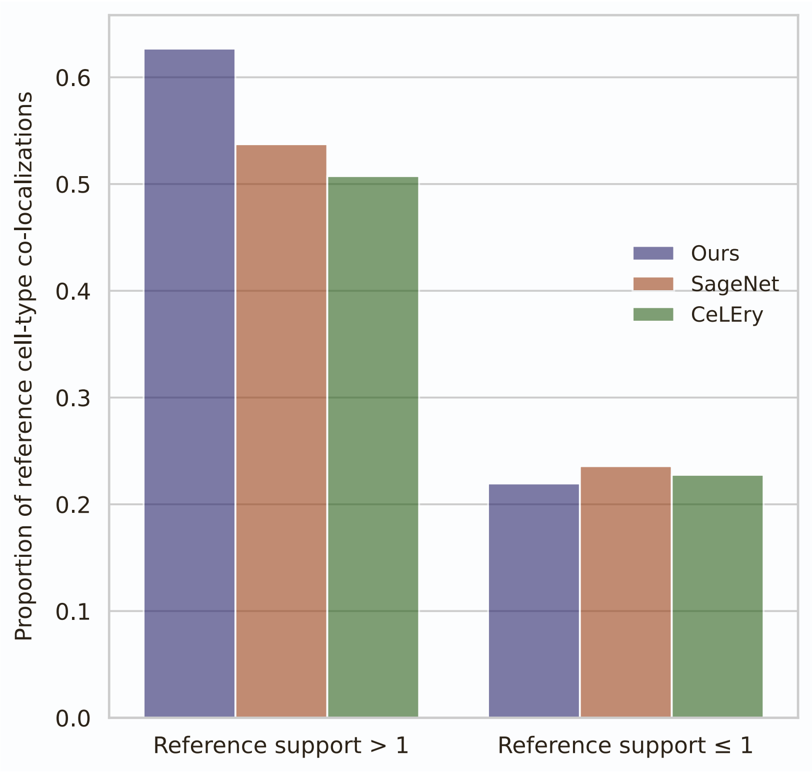

Figure S13. Detected co-localization proportions in the SC Sample vs. Reference Patterns. The SC Sample was spatially reconstructed for the S37 from the mouse scRNA-seq atlas, which was at the same developmental stage (E8.5) with the 6 reference ST samples.

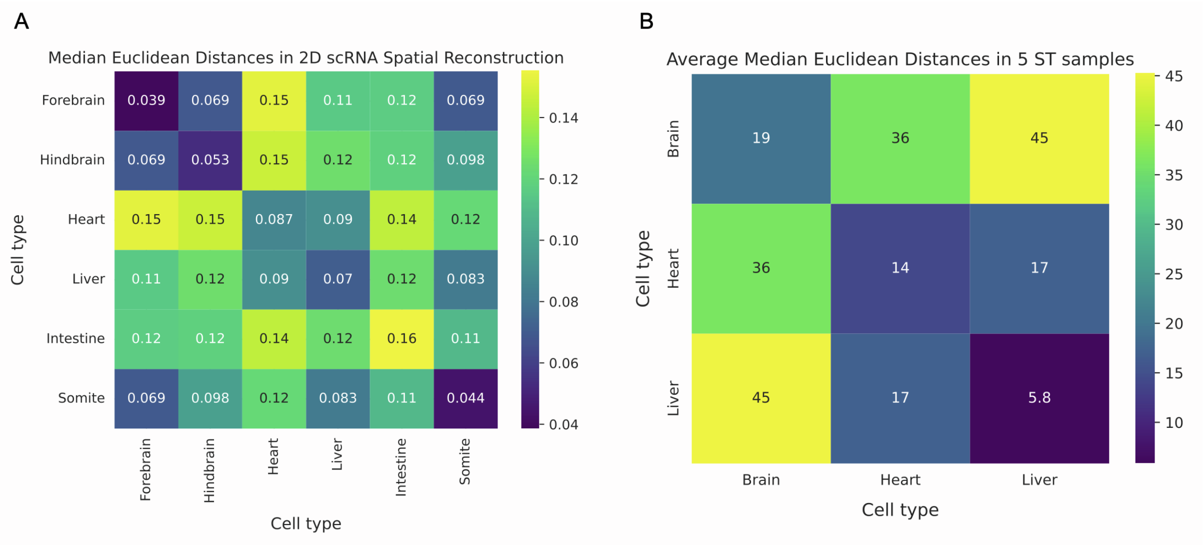

Figure S14. Distances of the cell-type pairs, that were calculated between all cells belonging to different cell types, using the median value to represent these cell-type distances. A, The predicted pairwise distances of cell-types for spatially reconstructed scRNA sample. B, The average pairwise distances between cell types for the five ST reference samples.

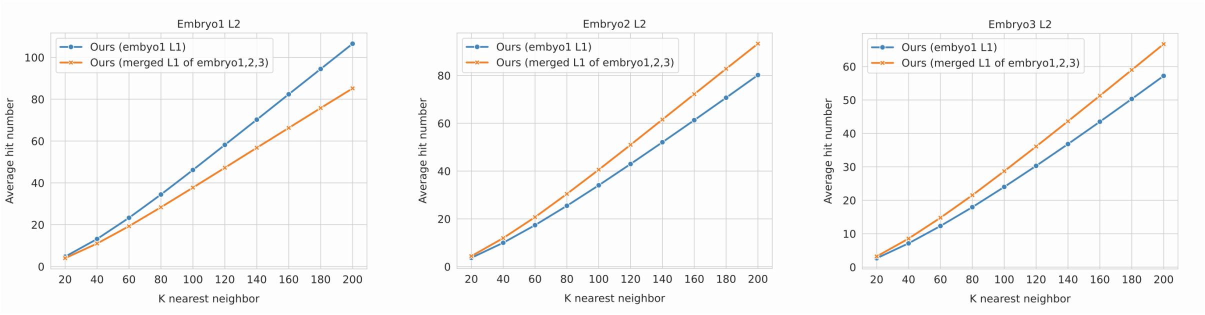

Figure S15. Evaluation of cell neighbor identification with multiple ST training samples.

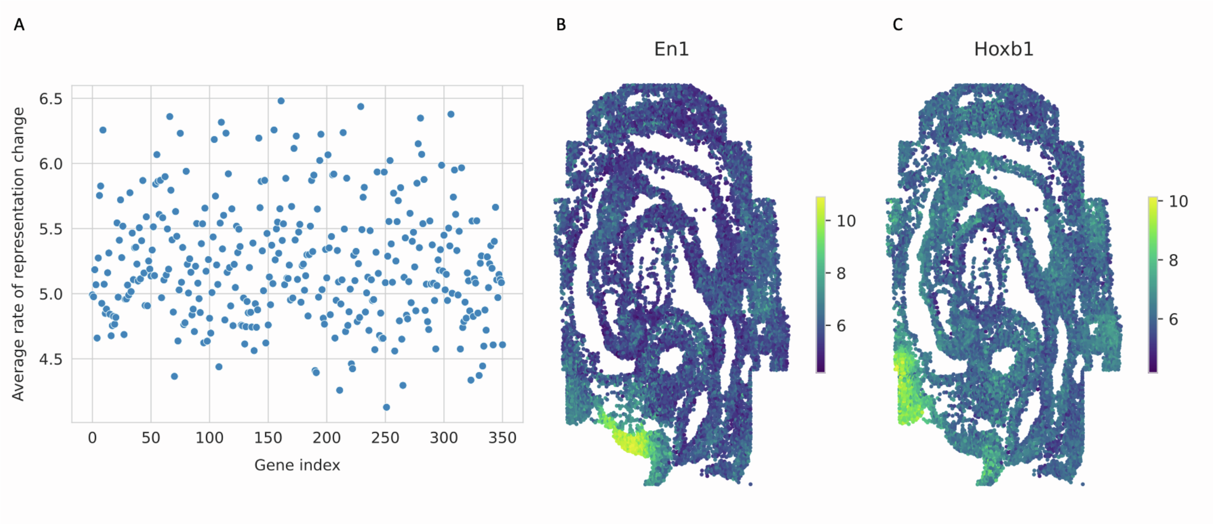

Figure S16. Analysis of spatially related genes. A, the distribution of average rate of representation changes for all genes. B, the rate of representation changes for the En1 gene in the Embryo1 L1 of SeqFISH data. Each dot in the plot represents an individual cell, with higher values indicating a greater contribution of the En1 gene to the spatially related representation of that cell. C, the rate of representation changes for the Hoxb1 gene in the Embryo1 L1 of SeqFISH data.

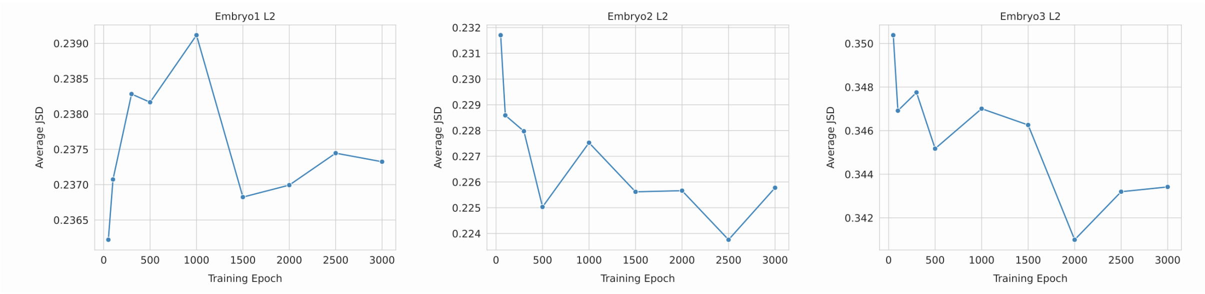

Figure S17. Impact of Training Epochs on Spatial Reconstruction Performance. This experiment was conducted for the evaluation of training epochs in the context of spatial reconstruction for mouse gastrulation cells using single-cell ST reference (as shown in Figure 2A of the main text).

A

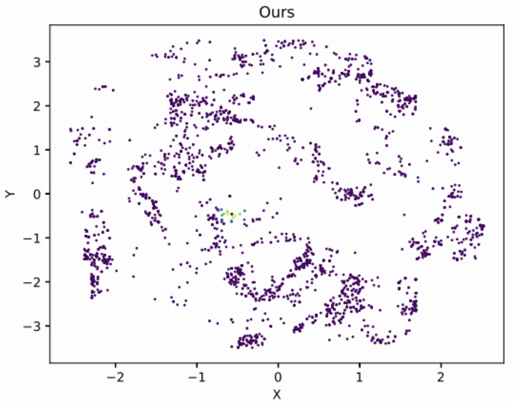

B

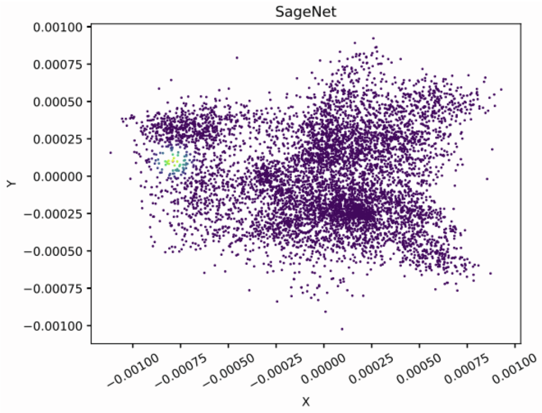

C

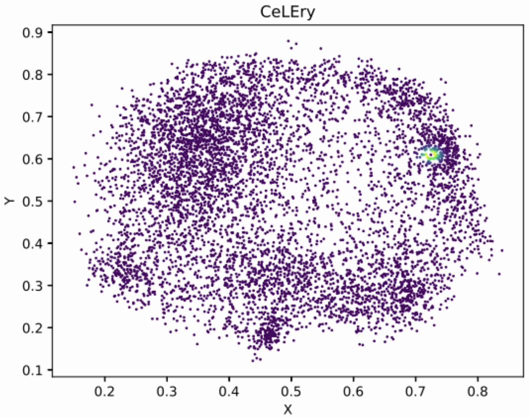

Figure S18. Weight matrix of random cell's neighbors for cell-type co-localization analysis using spatialDM. A, Weight matrix based on our method's reconstructed locations. B, Weight matrix based on SageNet's reconstructed locations. C, Weight matrix based on by CeLEry's reconstructed locations.

2. Supplementary Tables

Table S1. Average spearman's rank correlation coefficient on the mouse right brain cells derived by MERSCOPE.

Method	Spearman's ρ
**Ours**	**0.766**
SageNet	0.492
CeLEry	0.672
novoSparc	0.341
Tangram	0.137

Table S2. Average spearman's rank correlation coefficient on the human lung samples derived by 10X Visium.

Method	Spearman's ρ
Ours	0.117
SageNet	0.172
CeLEry	0.009
novoSparc	0.029
Tangram	0.006
Null	0

Table S3. Contingency table of detected cell-type co-localizations in reference datasets and SC sample that spatially reconstructed using our method.

Detected co-localizations in reference datasets	Detected co-localizations in SC sample	
	Detected	Not detected
Reference support > 1	42	25
Reference support ≤ 1	23	100

Table S4. Contingency table of detected cell-type co-localizations in reference datasets and SC sample that spatially reconstructed using CeLEry.

Detected co-localizations in reference datasets	Detected co-localizations in SC sample	
	Detected	Not detected
Reference support > 1	42	25
Reference support ≤ 1	35	88

Table S5. Contingency table of detected cell-type co-localizations in reference datasets and SC sample that spatially reconstructed using SageNet.

Detected co-localizations in reference datasets	Detected co-localizations in SC sample	
	Detected	Not detected
Reference support > 1	38	29
Reference support ≤ 1	26	97

Table S6. Average spearman's rank correlation coefficient for all benchmarking scenarios by setting the *m* as 21 (Eq. 3 in the Methods).

Training ST dataset	Testing dataset	Spearman's ρ
SeqFISH mouse embryo1 L1	SeqFISH mouse embryo1 L2	0.89
	SeqFISH mouse embryo2 L2	0.634
	SeqFISH mouse embryo3 L2	0.532
Stereo-Seq mouse embryo	SeqFISH mouse embryo1 L2	0.249
	SeqFISH mouse embryo2 L2	0.464
	SeqFISH mouse embryo3 L2	0.288
10X Visium human breast S06	10X Visium human breast S08	0.1
MERSCOPE left mouse brain	MERSCOPE right mouse brain	0.775

Table S7. Average JSD (k=20) and Spearman's correlation coefficient on using 3 samples, including L1 of embryo1,2,3 as the reference for spatial reconstruction of mouse gastrulation cells.

Method	Embryo1 L2		Embryo2 L2		Embryo3 L2	
	JSD	Spearman's ρ	JSD	Spearman's ρ	JSD	Spearman's ρ
Ours (embryo1 L1)	0.237	0.874	0.225	0.574	0.343	0.477
Ours (merged L1 of embryo1,2,3)	0.254	0.846	0.212	0.591	0.324	0.422

Table S8. Runtime and memory usage for fitting CellContrast model.

Method	Fitting time	Average memory	Peak memory
Ours (CPU)	8:28:10	0.789GB	5.264GB
Ours (GPU)	2:17:19	2.455GB	2.457GB

*Note: the training epoch is 3000. All experiments were conducted on a machine with 24 cores using SeqFISH embryo1 L1 (10,150cells) as the training sample.
